# A Systematic Review of the Potential Chemoprotective Effects of Resveratrol on Doxorubicin-Induced Cardiotoxicity: Focus on the Antioxidant, Antiapoptotic, and Anti-Inflammatory Activities

**DOI:** 10.1155/2021/2951697

**Published:** 2021-08-22

**Authors:** Li-Feng Hu, Huan-Rong Lan, Xue-Min Li, Ke-Tao Jin

**Affiliations:** ^1^Department of Colorectal Surgery, Shaoxing People's Hospital (Shaoxing Hospital, Zhejiang University School of Medicine), Shaoxing, Zhejiang 312000, China; ^2^Department of Breast and Thyroid Surgery, Affiliated Jinhua Hospital, Zhejiang University School of Medicine, Jinhua, 321000 Zhejiang Province, China; ^3^Department of Hepatobiliary Surgery, Affiliated Jinhua Hospital, Zhejiang University School of Medicine, Jinhua, 321000 Zhejiang Province, China; ^4^Department of Colorectal Surgery, Affiliated Jinhua Hospital, Zhejiang University School of Medicine, Jinhua, 321000 Zhejiang Province, China

## Abstract

**Purpose:**

Although doxorubicin chemotherapeutic drug is commonly used to treat various solid and hematological tumors, its clinical use is restricted because of its adverse effects on the normal cells/tissues, especially cardiotoxicity. The use of resveratrol may mitigate the doxorubicin-induced cardiotoxic effects. For this aim, we systematically reviewed the potential chemoprotective effects of resveratrol against the doxorubicin-induced cardiotoxicity.

**Methods:**

In the current study, a systematic search was performed based on Preferred Reporting Items for Systematic Reviews and Meta-Analyses (PRISMA) guideline for the identification of all relevant studies on “the role of resveratrol on doxorubicin-induced cardiotoxicity” in the electronic databases of Web of Science, PubMed, and Scopus up to March 2021 using search terms in their titles and abstracts. Two hundred and eighteen articles were screened in accordance with a predefined set of inclusion and exclusion criteria. Finally, 33 eligible articles were included in this systematic review.

**Results:**

The *in vitro* and *in vivo* findings demonstrated a decreased cell survival, increased mortality, decreased heart weight, and increased ascites in the doxorubicin-treated groups compared to the control groups. The combined treatment of resveratrol and doxorubicin showed an opposite pattern than the doxorubicin-treated groups alone. Furthermore, this chemotherapeutic agent induced the biochemical and histopathological changes on the cardiac cells/tissue; however, the results (for most of the cases) revealed that these alterations induced by doxorubicin were reversed near to normal levels (control groups) by resveratrol coadministration.

**Conclusion:**

The results of this systematic review stated that coadministration of resveratrol alleviates the doxorubicin-induced cardiotoxicity. Resveratrol exerts these chemoprotective effects through several main mechanisms of antioxidant, antiapoptosis, and anti-inflammatory.

## 1. Introduction

Cancer, the uncontrolled growth of cells, is the second leading cause of death in the world [1–3], and also, its incidence and mortality are rapidly growing [4]. According to a recent report, an estimated 19.3 million new cancer cases and almost 10.0 million cancer deaths happened worldwide in 2020 [5]. The conventional modalities for cancer treatment are surgery, chemotherapy, and radiotherapy [6–9]. Although chemotherapy is effectively used for systemic treatment of different cancers, it suffers from several restrictions, such as nonspecificity and various adverse effects on normal cells and tissues; hence, its clinical utility is limited [10, 11].

Doxorubicin (Adriamycin) belongs to the anthracycline class medication which is commonly applied since the late 1960s for treatment of various tumors, including Hodgkin lymphoma, non-Hodgkin lymphoma, breast cancer, lung cancer, testicular cancer, thyroid cancer, and ovarian cancer [12–14]. Some immediate side effects have been reported for this chemotherapeutic drug, which are reversible or clinically manageable, such as arrhythmia, myelosuppression, vomiting, and nausea [13]. However, doxorubicin-induced cardiotoxicity is a serious adverse effect and can reduce quality of life and sometimes fatalities, leading to restriction of the clinical use of this chemotherapy drug [15]. In this regard, the use of chemoprotective agents during doxorubicin treatment has been suggested which may mitigate the adverse effects and improve patient survival.

**Figure 1:**
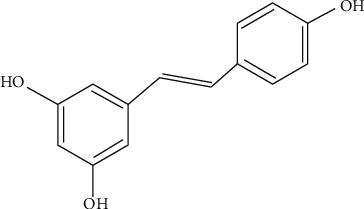
Chemical structure of resveratrol.

According to the published studies, it can be mentioned that using the herbal and natural products or their derivatives to alleviate the chemotherapy-induced adverse effects (chemoprotectors) or increase the sensitivity of cancer cells to chemotherapy drugs (chemo/radiosensitizers) has attracted much attention over the past several decades. Resveratrol (3,5,4′-trihydroxy-trans-stilbene, Figure 1), as a natural polyphenol, is found in more than 70 plant species. The main sources reported for this herbal agent are grapes, red wine, soy, and peanuts [16–19]. Naturally, resveratrol can protect the herbs against fungal, ultraviolet rays, and other stresses [20]. Moreover, it is reported that this herbal agent has potent antioxidant and anticlastogenic activities, helping to protect against genomic instability and carcinogenesis [21, 22]. Resveratrol also has some abilities to kill tumoral cells and sensitize tumor cells to therapeutic modalities such as chemotherapy and radiotherapy [21], as its anticancer activity has been assessed in many tumor types, such as colorectal cancer, prostate cancer, lung cancer, liver cancer, and breast cancer [23–26]. Additionally, it has been shown that resveratrol not only acts as a chemosensitizer agent but also has chemopreventive activities which are linked to its antiproliferative, antioxidant, antiapoptosis, and anti-inflammatory activities [27, 28]. Other biological activities of resveratrol such as neuroprotective, radioprotective, and cardioprotective properties have also been reported [19, 29]. It has also recently been reported that resveratrol can be effective in the management of acute pancreatitis [30].

To the best of our knowledge, this study is the first systematic review on the cardioprotective role of resveratrol, as an adjuvant, during doxorubicin treatment. Additionally, it was tried to discuss the following questions. (1) How does doxorubicin chemotherapeutic agent lead to cardiotoxicity? (2) What are the underlying mechanisms of doxorubicin-induced cardiotoxicity? (3) What is the role of resveratrol on the doxorubicin-induced cardiotoxicity? (4) What are the cardioprotective mechanisms of resveratrol against doxorubicin-induced cardiotoxicity?

## 2. Methods

The present systematic review was performed based on the Preferred Reporting Items for Systematic Reviews and Meta-Analyses (PRISMA) guidelines [31] (see Table 1). A PICO framework was also applied to structure the review process [31] that was composed of participants (P): doxorubicin-damaged cardiac cells (in vitro studies) and/or patients/animals with doxorubicin-induced cardiotoxicity (clinical/in vivo studies); intervention (I): chemotherapies with doxorubicin-based regimens; comparison (C): patients/animals/cells treated with resveratrol and doxorubicin; and outcomes (O): we selected two critical outcomes as follows: (i) changes in the cardiac cells/tissue following doxorubicin administration compared to control or untreated groups and (ii) changes in the cardiac cells/tissue following combined treatment of resveratrol and doxorubicin compared to doxorubicin treatment alone. Of note, the review protocol was not registered.

### 2.1. Search Strategy

A comprehensive systematic search was performed to obtain all relevant studies on “the role of resveratrol on doxorubicin-induced cardiotoxicity” in both medical subject heading (MeSH) or advance in the electronic databases of Web of Science, PubMed, and Scopus up to Mach 2021 using the keywords “Resveratrol” AND “Doxorubicin” OR “Adriamycin” AND “Heart” OR “myocardium” OR “Myocardial” OR “Cardiac Toxicity” OR “Cardiac Toxicities” “Cardiomyopathy” OR “Myocyte” OR “Cardiopathic” OR “Cardiopathy” OR “cardiotoxicity” OR “Cardiotoxicities” OR “Cardiomyocyte” OR “Arrhythmias” OR “Cardiac” in title, abstract, or keywords.

### 2.2. Study Selection Process

The inclusion criteria considered in the current study were full-text articles with (a) English language, (b) our per-defined purpose on the role of resveratrol on doxorubicin-induced cardiotoxicity (based on the aforementioned keywords), (c) adequate data, (d) no restriction in publications with clinical, in vivo, or in vitro studies, and (e) no restriction on publication year. The exclusion criteria considered for this study were (a) hemodynamic data, (b) not related studies, (c) review articles, (d) case reports, (e) editorials, (f) letters to the editors, (g) oral presentations, (h) posters, and (i) book chapters.

### 2.3. Data Extraction Process

Each eligible study was assessed by two researchers and the following information were then extracted: (1) author name and year of publication, (2) models (clinical, in vivo, or/and in vitro), (3) doxorubicin dosage, protocol of usage, and type of administration route, (4) outcomes of doxorubicin on cardiac cells/tissue, (5) resveratrol dosage, protocol of usage, and type of administration route, and (6) resveratrol coadministration outcomes.

## 3. Results

### 3.1. Literature Search and Screening

**Figure 2:**
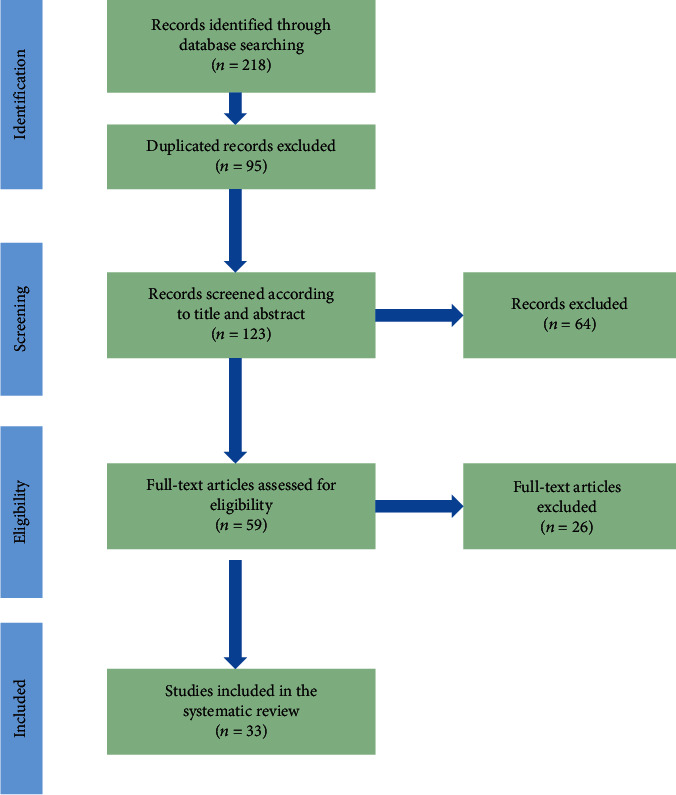
Flow diagram of PRISMA used in the present systematic review for selection process.

In Figure 2, the process of study selection is shown.

Two hundred and eighteen articles were obtained by a systematic search on the above-mentioned electronic databases up to March 2021. After removing the duplicated articles (*n* = 95), the remaining ones (*n* = 123) were screened in their titles and abstracts, and 64 of them were excluded. Fifty-nine articles were qualified for evaluation of their full texts. Thirty-three articles were finally included in the current study based on the inclusion and exclusion criteria. A summary of the data extracted from the eligible articles are listed in Table 2.

### 3.2. The Role of Resveratrol on Doxorubicin-Induced Cardiotoxicity

#### 3.2.1. Cell Viability and Survival Study

The *in vitro* findings revealed that the cell survival following treatment with doxorubicin was significantly less than the control group [32–43]. It was also fund that the doxorubicin-mediated cytotoxicity in cardiac cells had time- and dose-dependent manners. The results showed that there was a direct relation between the decreased cell count and posttreatment time/chemotherapy dosage [32, 34, 37, 39, 42]. However, the data obtained from the cell viability assay demonstrated that cotreatment of cardiac cells with resveratrol resulted in significant protective effects against doxorubicin-induced decrease in cell viability [32–43].

According to the *in vivo* results, the mortality of mice/rats treated with doxorubicin was significantly higher than that of the untreated group. The use of resveratrol significantly decreased doxorubicin-induced mortality [38, 44, 45]. For instance, Angelis et al. reported that the mortality rate of 67% observed in doxorubicin-treated animals was reduced to 33% in the group cotreated with resveratrol and doxorubicin [38]. In other study by Cappetta et al., the mortality rate of 40% observed in doxorubicin-treated rats was declined to 12% in the resveratrol plus doxorubicin group [44]. Furthermore, it was found that resveratrol cotreatment delayed the doxorubicin-induced mortality in mice/rats to a much greater extent than the chemotherapy group alone [38, 44, 45].

#### 3.2.2. Body Weight, Heart Weight, and Ascites Changes

The results demonstrated that the body weight and heart weight of mice/rats were reduced in the doxorubicin groups than in the control groups [38, 43, 45–52]. Additionally, it was observed that the ratio of heart to the body weight as well as the ratio of heart weight to tibia length of animals was decreased following doxorubicin administration [43, 47, 48, 50]. Moreover, there was a significant accumulation of ascites in the doxorubicin-treated rats compared to the untreated rats. Of note, the survival rate in the rats with high ascites was significantly more than the other rats [38].

Coadministration of resveratrol and doxorubicin to the mice/rats increased the body weight, heart weight, ratio of heart to body weight, and ratio of heart weight to tibia length compared to the doxorubicin-treated groups alone [38, 43, 45–49, 51, 52]. The increased ascites values of doxorubicin-treated rats were significantly decreased by resveratrol cotreatment [38].

#### 3.2.3. Changes Induced in Biochemical Markers

Some studies showed that the use of doxorubicin chemotherapy drug can induce the biochemical changes on heart cells/tissue (listed in Table 2). Briefly, it was observed that the reactive oxygen species (ROS), aspartate aminotransferase (AST), triglycerides, total cholesterol, 8-OHdG, malondialdehyde (MDA), myeloperoxidase (MPO), thiobarbituric acid reactive substances (TBARS), protein carbonyl, phosphor-p38, p53, p300, Bcl-2-associated X protein (BAX), cleaved caspase-3, cleaved poly (ADP-ribose) polymerase (PARP), atrial natriuretic peptide, fatty acid binding protein, creatine phosphokinase (CPK), creatine kinase-myocardial band (CK-MB), transforming growth factor beta 1 (TGF-*β*1), E2F transcription factor 1 (E2F1), adenosine monophosphate- (AMP-) activated protein kinase (AMPK) *α*2, myocardial collagen I mRNA, collagen I/III, matrix metalloproteinase-2 (MMP-2), fibronectin, LC3-II, Beclin-1, nuclear factor of activated T cells 3 (NFAT3), mammalian target of rapamycin complex 1 (mTORC1), serum troponin-I, toll-like receptor-4 (TLR-4), interleukin-6 (IL-6), tumor necrosis factor alpha (TNF-*α*), and inducible nitric oxide synthase (iNOS) levels were significantly increased following doxorubicin administration than the control groups [35–38, 40–44, 46–61]. In contrast, the glutathione (GSH), catalase, superoxide dismutase (SOD), manganese SOD (MnSOD), glutathione peroxidase (GPx), alkaline phosphatase, GSH to glutathione disulfide (GSSG) ratio, total antioxidant capacity, B-cell lymphoma-extra large (Bcl-xL), B-cell lymphoma 2 (Bcl-2), heme oxygenase-1 (HO-1), phospho-AKT, insulin-like growth factor 1 receptor (IGF-1R), LC3-II/LC3-I, phosphor-AMPK, NFAT5, vascular endothelial growth factor B (VEGF-B), and phospho-glycogen synthase kinase 3 beta (GSK-3*β*) levels were significantly decreased in the doxorubicin-treated groups compared to the control groups [38, 40, 41, 43, 46, 48–51, 53–59, 61].

The resveratrol cotreatment alleviated doxorubicin-induced biochemical changes on heart cells/tissue (for most of the cases) [35–38, 40–44, 46–61].

Some studies have reported conflicting findings on several biochemical markers (see Table 2). For instance, several studies demonstrated the elevated levels of lactate dehydrogenase (LDH) [41, 43, 46–48, 51, 53, 54, 61], creatine kinase [48, 58], and sirtuin 1 (SIRT1) [39, 47] following doxorubicin treatment alone, while other studies showed the decreased levels for these biomarkers [35, 38, 41, 44, 55, 58, 59]. Nevertheless, the combined treatment of resveratrol and doxorubicin showed a reverse manner on these biomarkers compared with chemotherapy groups alone [35, 38, 41, 43, 44, 46–48, 51, 53, 54, 58, 59].

#### 3.2.4. Histological Changes

A series of histological changes in cardiac tissue of animals have been reported following doxorubicin treatment, including congestion of blood vessels, cytoplasmic vacuolization, increments in edema, necrosis and inflammatory infiltration, hyalinization of muscle fiber, chromatin margination of nuclei and pyknotic nuclei, massive fragmentation and lysis of myofibrils, complete loss of cristae, interruption of Z lines, myocyte wavy degeneration, interstitial fibrosis, massive deposition of collagen, widely spaced deep acidophilic fibers, dense collagen fibers among thin fibers, massive interstitial hemorrhage and interfibrillar congestion, and so on (the other histological changes are represented in Table 2) [45, 46, 49, 51, 53–55, 62, 63].

According to the results of most studies, it was found that resveratrol coadministration can alleviate the doxorubicin-induced histological changes [45, 46, 49, 51, 54, 55, 62, 63].

## 4. Discussion

**Figure 3:**
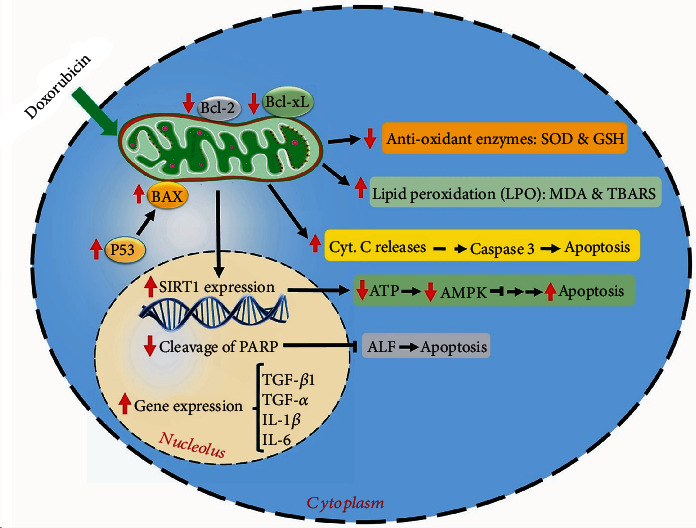
The molecular mechanisms of doxorubicin-induced cardiac cytotoxicity. This chemotherapy agent induces oxidative stress mostly via mitochondrial dysfunction. Doxorubicin increases free radicals via inhibition of SOD and GSH enzymes and also elevates LPO markers (MDA and TBARS). Moreover, doxorubicin increases apoptosis via reductions in BCL-2 and Bcl-xL, increments in BAX and p53 activations, increment in cytochrome C release, and elevation in caspase-3 level. Additionally, it induces apoptosis via reduction of PARP cleavage, as it leads to reductions in ATP and AMPK levels. Furthermore, doxorubicin increases the inflammatory mediators (such as IL-6, IL-1*β*, TNF-*α*, and TGF-*β*1), leading to cell injury. Resveratrol, through an opposite pattern (antioxidant, antiapoptotic, and anti-inflammatory activities), mitigates doxorubicin-induced cardiac cytotoxicity. ↑: increased by doxorubicin; ↓: decreased by doxorubicin; AMPK: AMP-activated protein kinase; AIF: apoptosis-inducing factor; Bcl-xL: B-cell lymphoma-extra large; Bcl-2: B-cell lymphoma 2; BAX: Bcl-2-associated X protein; GSH: glutathione; IL-6: interleukin-6; LPO: lipid peroxidation; MDA: malondialdehyde; SOD: superoxide dismutase; SIRT1: sirtuin 1; PARP: poly (ADP-ribose) polymerase; TBARS: thiobarbituric acid reactive substances; TGF-*β*1: transforming growth factor beta 1; TNF-*α*: tumor necrosis factor alpha.

In the present systematic review, we aimed to assess the adverse effects of doxorubicin chemotherapeutic drug on the cardiac cells/tissue. The coadministration effects of resveratrol on this disorder were also investigated. Table 2 represents a summary of these findings. Furthermore, some of the important alterations on the cardiac cell following doxorubicin treatment as well as the effects of resveratrol on these changes are depicted in Figure 3.

Anthracyclines, as a class of chemotherapy drugs, can lead to chronic toxicity to the heart. The cardiotoxicity is more common in patients receiving doxorubicin, the most familiar anthracycline [64]. This chemotherapy agent has been widely applied for over forty years in case of different hematological and solid cancers [65]. In the cancerous cells, doxorubicin is capable of intercalation into DNA and disruption of DNA repair mediated by topoisomerase-II generation as well as production of free radicals and their damage to proteins, DNA, and cellular membranes [66]. Although doxorubicin has been standardized as an antitumoral drug, its potential life-threatening cardiomyopathy and congestive cardiac failure should be taken into consideration [67]. It has been shown that doxorubicin exerts toxic effects through oxidative stress, apoptosis, inflammation activities, etc. [68]. To mitigate the doxorubicin-induced cardiotoxicity, various studies have shown that the use of chemoprotective agents can provide acceptable results. In this regard, the combination treatment of doxorubicin with less toxic substances derived from plants, such as resveratrol, has received more attention in recent years.

The antitumoral activity of resveratrol has been reported in some cancers [69–72]. Resveratrol can also affect the expression of different genes [73, 74]. Additionally, it has been shown that resveratrol cotreatment not only can alleviate chemotherapy-induced adverse effects but can also decrease drug resistance (synergistic effect) [75, 76]. Resveratrol exerts its chemoprotective effects via several mechanisms, including antioxidant, antiapoptotic, and anti-inflammatory activities. In the following, the mechanistic effects of doxorubicin chemotherapeutic drug on the cardiac cellular pathway and also the chemoprotective effects of resveratrol and its underlying mechanisms on the doxorubicin-induced cardiotoxicity are discussed.

### 4.1. Antioxidant Actions

Although oxygen molecules are necessary to survive aerobic cells, their forms of free radicals are dangerous to the cells [77]. On the other hand, the free radicals are commonly generated in the cells, but they are neutralized by several defense mechanisms [78]. In oxidative stress conditions, such as chemotherapy, the amount of free radicals increases and an imbalance is created between the generated free radicals and the antioxidant defense system [79, 80]. It has been reported that the use of doxorubicin increases the ROS level of cardiac cells, and subsequently, they are able to attack the cell macromolecules, leading to malfunction of the heart tissue [35, 38, 50, 59]. Upon mitochondrial injury, the generation of free radicals in the cells elevates [81]; in this regard, doxorubicin through impairment of mitochondrial malfunction can increase the generated free radicals [35]. This chemotherapeutic agent also increases lipid peroxidation (LPO) markers of MDA and TBARS in the heart tissue, leading to the cell membrane devastation and malfunction [41, 46, 47, 49, 53–55, 58, 61]. Furthermore, it has been reported that doxorubicin administration declines GSH, catalase, GPx, and MnSOD levels [41, 46, 49, 50, 53–55, 58, 61]. Of note, H_2_O_2_ (as a nonradical ROS) through the activity of GPx enzyme and consuming GSH produces 2H_2_O [82]. The catalase enzyme also decomposes H_2_O_2_ to H_2_O and O_2_ [83]. Additionally, MnSOD is the mitochondrial SOD and acts as the primary line of defense system against oxidant stress [84]. These findings represent that doxorubicin impairs scavenging capacity of intracellular antioxidant enzymes.

According to the obtained results, it was found that resveratrol via the antioxidant actions could decrease doxorubicin-elevated ROS level of cardiac cells [35, 38, 50, 59]. This antioxidant agent is also able to polarize mitochondria, thereby inhibiting doxorubicin-induced ROS generation [35]. Furthermore, it has been reported that increased MnSOD activity following resveratrol cotreatment may play a main role in the reduction of ROS because the mitochondrion is the site of doxorubicin accumulation and the associated ROS generation [35, 50, 85]. Moreover, this herbal agent could upregulate GSH, catalase, and GPx expressions [38, 41, 44, 46, 49, 53, 54, 58, 61] and downregulate MDA and TBARS levels in the cardiac cells [41, 46, 47, 49, 53, 54, 58].

### 4.2. Antiapoptotic Actions

Apoptosis, as a physiological pathway, regulates cell death and controls the cell numbers. It is also needed to eliminate the harmed or transformed cells [86, 87]. The oxidative stress conditions and massive DNA injury can lead to apoptosis [88, 89]. There are some important mediators which are involved in the apoptosis process, including caspase enzymes, PARP, Bcl-2, p53, BAX, Bcl-xL, NFAT5, and ceramide [15, 90–99]. Evasion of apoptosis is one of the features of most cancerous cells, because any irregularity in this physiological process can induce cancer. Some chemotherapeutic agents are aimed at inducing apoptosis in cancerous cells [100]. It has been reported that doxorubicin administration increases apoptosis levels in the cardiac cells than the control groups [38–41, 43, 44, 47, 48, 56, 57, 59, 60, 101]. It is noteworthy that doxorubicin-induced apoptotic cardiomyocyte death (as a pathogenic mechanism in acute cardiotoxicity) [79, 102] and mitochondrial-dependent intrinsic apoptosis are considered as the main reasons for cardiac dysfunction [103]. Doxorubicin can downregulate Bcl-xL expression [51] and also upregulate BAX, cleaved caspase-3, and p53 expressions in the cardiac cells compared to the untreated groups [36–38, 40–44, 47–49, 51, 56, 57, 59, 60]. These findings indicate that the cells are moving towards apoptosis. Furthermore, doxorubicin can elevate PARP activity [36, 37, 42]. PARP is a nuclear enzyme and can regulate many cellular processes such as DNA repair, apoptosis, chromatin functions, and genomic stability [15, 104]. Doxorubicin has also been implicated to trigger cardiac apoptosis through activation of c-Jun N-terminal kinase (JNK), p38, and p53 mitogen-activated protein kinase (MAPK) pathways [105]. MAPKs may also affect NFAT5 [106–109]. NFAT5 is a transcription factor which has a critical role in apoptosis [99]. It was shown that doxorubicin led to the increased level of NFAT5 in the cardiac cells [51].

Some studies have reported that resveratrol is able to induce apoptosis in different cancerous cells [110–115]. In addition to its apoptotic activities, this herbal agent through antiapoptotic effects can protect normal cells/tissues. According to the data represented in the current study, it was shown that combined treatment of resveratrol and doxorubicin declines the apoptosis level of cardiac cells compared to the doxorubicin-treated groups alone [38–41, 43, 44, 47, 48, 56, 57, 59, 60]. Resveratrol combined to doxorubicin also upregulated Bcl-xL expression in the cardiac cells treated by doxorubicin [51], while this antiapoptotic agent downregulated BAX, cleaved caspase-3, p53, and PARP expressions [36–38, 40–44, 47–49, 51, 56, 57, 59, 60]. Furthermore, resveratrol cotreatment suppressed the increased levels of phospho-p38 and NFAT5 in the doxorubicin-treated cardiac cells [51, 52, 57, 59].

### 4.3. Anti-Inflammatory Actions

The inflammatory process can occur following tissue injuries induced from various harmful stimuli, including chemotherapy, radiotherapy, microbial pathogen infection, and/or wounding [116–119]. The inflammation plays a vital role in tumor resistance and is responsible for the incidence of different adverse effects following chemotherapy [120]. It has been reported that doxorubicin can induce heart inflammation during cancer chemotherapy [53, 55]. Moreover, doxorubicin-induced oxidative stress can affect LPO and activate lysosomal enzymes, leading to promote the inflammation in heart tissue [15]. It has also been reported that the doxorubicin administration upregulates proinflammatory mediators of TNF-*α*, intercellular adhesion molecule-1 (ICAM-1), cyclooxygenase-2 (COX-2), TGF-*β*, nuclear factor-kappa B (NF-*κ*B), MPO, IL-1*β*, and IL-6 levels in the cardiac cells [44, 46, 49, 53, 121]. ICAM-1 is a surface protein that is able to infiltrate leucocytes to the damaged regions of heart tissue [122]. COX-2 is a proinflammatory enzyme, and it is overexpressed at the inflammatory site of cancer [123]. TGF-*β*, as a profibrogenic cytokine, mediates several aspects of the fibrotic process. In this regard, it induces fibroblast proliferation and transformation to myofibroblasts which leads to the deposition of collagen and extracellular matrix protein [124, 125]. TGF-*β* is also able to modulate cell proliferation, differentiation, apoptosis, and migration [126]. Furthermore, doxorubicin could decline HO-1 level in the cardiac cells [48]. HO-1 is a nuclear factor-erythroid factor 2-related factor 2- (Nrf2-) regulated gene that has a vital role in the prevention of vascular inflammation [127].

The anti-inflammatory effects of resveratrol on various normal/tumoral tissues have been reported [22, 128–132]. Resveratrol, through its anti-inflammatory activities, can protect the normal cells and also decrease the resistance of cancer cells to chemotherapy drugs. The findings represented in the current systematic review demonstrated that resveratrol cotreatment alleviates the doxorubicin-induced cardiac inflammation. In this regard, it was found that combined treatment of resveratrol and doxorubicin declines the elevated levels of TNF-*α*, TGF-*β*1, MPO, and IL-6 and also increased HO-1 level in the heart tissue of the doxorubicin-treated rats [44, 46, 49, 53, 57]. Moreover, the histological findings showed that doxorubicin-induced inflammation is mitigated by resveratrol administration [46, 49, 53, 55, 58].

## 5. Perspective of Future Research

Doxorubicin, as a chemotherapy drug, is commonly used for the treatment of various cancers. Nevertheless, the clinical use of doxorubicin may be restricted because of its adverse effects on the normal cells/tissues, especially cardiotoxicity. Researchers reported that the use of chemoprotective agents, such as resveratrol, can alleviate the doxorubicin-induced cardiotoxicity. This herbal agent exerts its chemoprotective effects through several main mechanisms of antioxidant, antiapoptosis, and anti-inflammatory. In addition to its chemoprotective effect, resveratrol can sensitize cancer cells to chemotherapy drugs (chemosensitizer effects) [133–136]. Despite its remarkable protective effects, unfavorable pharmacokinetics/pharmacodynamics profile of resveratrol such as poor bioavailability has restricted its applications [137, 138]. To solve this problem, some studies have introduced novel derivatives and analogues for this chemoprotective agent [139–147]. Additionally, it has been proposed that nanostrategies for delivery of resveratrol can overcome these limitations [20, 148–151].

It should be mentioned that the data represented in the current systematic review are based on *in vitro* and *in vivo* models. Therefore, suggesting the use of resveratrol as a chemoprotector agent combined to doxorubicin in cancer patients requires further studies, because sometimes results are different between the *in vitro* and *in vivo* models and clinical studies.

## 6. Limitations

Some limitations should be addressed. Firstly, significant heterogeneity was encountered perhaps because of different regimens, doses, duration, center settings, populations enrolled, and so on, calling for cautious interpretation of the findings. Secondly, many of the studies suffer from significant sources of bias. Thirdly, the effect in many occasions was evaluated by very few studies; therefore, the evidence to support it is low.

## 7. Conclusion

The findings showed that doxorubicin chemotherapeutic agent can induce the biochemical and histological changes on the cardiac cells/tissue. However, using resveratrol alleviates the doxorubicin-induced adverse effects on the cardiac cells/tissue. Mechanically, resveratrol exerts its chemoprotective effects through several main mechanisms of antioxidant, antiapoptosis, and anti-inflammatory.

## Figures and Tables

**Table 1:** PRISMA checklist [31].

Section/topic	#	Checklist item	Reported on page #
*Title*			
Title	1	Identify the report as a systematic review, meta-analysis, or both.	1
*Abstract*			
Structured summary	2	Provide a structured summary including, as applicable, background; objectives; data sources; study eligibility criteria, participants, and interventions; study appraisal and synthesis methods; results; limitations; conclusions and implications of key findings; systematic review registration number.	2
*Introduction*			
Rationale	3	Describe the rationale for the review in the context of what is already known.	3 and 4
Objectives	4	Provide an explicit statement of questions being addressed with reference to participants, interventions, comparisons, outcomes, and study design (PICOS).	4
*Methods*			
Protocol and registration	5	Indicate if a review protocol exists, if and where it can be accessed (e.g., Web address), and, if available, provide registration information including registration number.	N/A
Eligibility criteria	6	Specify study characteristics (e.g., PICOS and length of follow-up) and report characteristics (e.g., years considered, language, and publication status) used as criteria for eligibility, giving rationale.	5 and 6
Information sources	7	Describe all information sources (e.g., databases with dates of coverage and contact with study authors to identify additional studies) in the search and date last searched.	5
Search	8	Present full electronic search strategy for at least one database, including any limits used, such that it could be repeated.	5
Study selection	9	State the process for selecting studies (i.e., screening, eligibility, included in systematic review, and, if applicable, included in the meta-analysis).	6
Data collection process	10	Describe method of data extraction from reports (e.g., piloted forms, independently, in duplicate) and any processes for obtaining and confirming data from investigators.	6
Data items	11	List and define all variables for which data were sought (e.g., PICOS and funding sources) and any assumptions and simplifications made.	6
Risk of bias in individual studies	12	Describe methods used for assessing risk of bias of individual studies (including specification of whether this was done at the study or outcome level) and how this information is to be used in any data synthesis.	N/A
Summary measures	13	State the principal summary measures (e.g., risk ratio and difference in means).	N/A
Synthesis of results	14	Describe the methods of handling data and combining results of studies, if done, including measures of consistency (e.g., *I*^2^) for each meta-analysis.	N/A
Risk of bias across studies	15	Specify any assessment of risk of bias that may affect the cumulative evidence (e.g., publication bias and selective reporting within studies).	N/A
Additional analyses	16	Describe methods of additional analyses (e.g., sensitivity or subgroup analyses and metaregression), if done, indicating which were prespecified.	N/A
*Results*			
Study selection	17	Give numbers of studies screened, assessed for eligibility, and included in the review, with reasons for exclusions at each stage, ideally with a flow diagram.	6-7 Figure 1
Study characteristics	18	For each study, present characteristics for which data were extracted (e.g., study size, PICOS, and follow-up period) and provide the citations.	7–10 Table 2
Risk of bias within studies	19	Present data on risk of bias of each study and, if available, any outcome level assessment (see item 12).	N/A
Results of individual studies	20	For all outcomes considered (benefits or harms), present for each study (a) simple summary data for each intervention group and (b) effect estimates and confidence intervals, ideally with a forest plot.	7–10 Table 2
Synthesis of results	21	Present results of each meta-analysis done, including confidence intervals and measures of consistency.	N/A
Risk of bias across studies	22	Present results of any assessment of risk of bias across studies (see item 15).	N/A
Additional analysis	23	Give results of additional analyses, if done (e.g., sensitivity or subgroup analyses and metaregression (see item 16)).	N/A
*Discussion*			
Summary of evidence	24	Summarize the main findings including the strength of evidence for each main outcome; consider their relevance to key groups (e.g., healthcare providers, users, and policy makers).	10–16
Limitations	25	Discuss limitations at study and outcome level (e.g., risk of bias) and at review level (e.g., incomplete retrieval of identified research and reporting bias).	16
Conclusions	26	Provide a general interpretation of the results in the context of other evidence and implications for future research.	16
*Funding*			
Funding	27	Describe sources of funding for the systematic review and other support (e.g., supply of data); role of funders for the systematic review.	17

**Table 2:** The characteristics of included studies.

Model	DOX dosage & protocol of usage; administration route	Outcomes of DOX on cardiac cells/tissue	Resveratrol dosage & protocol of usage; administration route	Resveratrol coadministration outcomes	Author & year
In vitro/H9c2 cells	5, 10, 20, 30, and 40 *μ*M & 24 h	↓Cell viability	100 *μ*M & 72 h prior to DOX treatment	↑Cell viability	Cao and Li, 2004 [32]
In vitro/neonatal rat ventricular myocytes	10 *μ*mol/L & 48 h	↓Cell viability	1 *μ*mol/L & cotreatment	↑Cell viability	Rezk et al., 2006 [33]
In vitro/H9c2 cells	20 *μ*mol/L & 4 h	↓Cell viability	5, 10, 20, and 40 *μ*M & 24 h prior to DOX treatment	↑Cell viability	Yu et al., 2007 [34]
In vitro/neonatal rat ventricular myocytes	1 and 50 *μ*M & 10 min and 24 h	↑ROS, ↓mitochondrial activity, ↑cell death, ↓JC-1 ratio (JC-1 aggregate/monomer), ↓SIRT1 activity (↑acetylated histone H3)	10 *μ*M & 72 h prior to DOX treatment	↓ROS, ↑mitochondrial activity, ↓cell death, ↓JC-1 ratio (JC-1 aggregate/monomer), ↑MnSOD, ↑SIRT1 activity (↓acetylated histone H3)	Danz et al., 2009 [35]
In vivo/rats	Cumulative dose of 20 mg/kg & for a 2-week period; *i.p.*	↓Body weight and heart weight, ↑plasma LDH activity, ↑CPK and AST levels, ↑total cholesterol and triglyceride levels, ↓total antioxidant capacity, ↑8-OHdG level, ↑luminol and lucigenin chemiluminescence levels, ↑MDA, ↓GSH, ↓SOD and catalase activities, ↑MPO, ↓Na^+^, K^+^-ATPase activity, ↑collagen content, congestion, and vacuolization in the cytoplasm of the cardiomyocytes	10 mg/kg/day & for 7 weeks (starting 2 weeks prior to DOX treatment); *i.p.*	↑Body weight and heart weight, ↓plasma LDH activity, ↓CPK and AST levels, ↓total cholesterol and triglyceride levels, ↑total antioxidant capacity, ↓8-OHdG levels, ↓luminol and lucigenin chemiluminescence levels, ↑luminol and lucigenin chemiluminescence levels, ↓MDA, ↑GSH, ↑SOD and catalase activities, ↓MPO, ↑Na^+^, K^+^-ATPase activity, ↓collagen content, ↓capillary vasocongestion, ↓vacuolization in the cytoplasm of the cells, regular cellular morphology	Tatlidede et al., 2009 [46]
In vivo/rats	4 mg/kg/day & for 1 week; *i.p.*	↑Creatine kinase, ↓LDH, ↑total cholesterol, ↑triglycerides, ↓GSH, GPx, SOD, and catalase activities, ↑TBARS, ↑edema	4 and 8 mg/kg/day & for 2 weeks (starting 1 week prior to DOX administration); enteral	↑LDH, ↓total cholesterol, ↓triglycerides, ↑GSH, GPx, SOD, and catalase activities, ↓TBARS, ↓edema	Mukherjee et al., 2011 [58]
In vivo/mice	Cumulative dose of 24 mg/kg & a single dose of 8 mg/kg at 3-week intervals; *i.p.*	↓Body weight, heart weight, and ratio of heart weight to body weight, ↑LDH, protein carbonyl content, and MDA levels, ↑apoptosis, ↑SIRT1 expression, ↑acetylation of p53, ↑binding activity of p53 to BAX promoter sequence, ↑BAX expression, ↑cytosolic concentration of cytochrome c, ↓release of cytochrome c from mitochondria	15 mg/kg/day & for 7 weeks; diet	↑Body weight, heart weight, and ratio of heart weight to body weight, ↓LDH, protein carbonyl content, and MDA levels, ↓apoptosis, ↑↑SIRT1 expression, ↓acetylation of p53, ↓binding activity of p53 to BAX promoter sequence, ↓BAX expression, ↓cytosolic concentration of cytochrome c, ↑release of cytochrome c from mitochondria	Zhang et al., 2011 [47]
In vivo/rats	1 and 2 mg/kg & once a week for seven weeks; *i.p.*	↑FABP and BNP levels (for 2 mg/kg), ↓creatine kinase, LDH, and ALP levels (for 2 mg/kg), ↑MDA+4HAE, ↓GSH/GSSG ratio (for 2 mg/kg), ↓SOD (for 2 mg/kg), induction of histological changes (↑interstitial edema, necrosis, and inflammatory infiltration)	20 mg/kg (of feed) & one week prior to DOX treatment + concomitantly with DOX until end of treatment; diet	↓FABP and BNP levels (for 2 mg/kg), ↓MDA+4HAE (for 1 mg/kg), ↓SOD (for 1 mg/kg), alleviation of DOX-induced histological changes	Dudka et al., 2012 [55]
In vivo/mice	Cumulative dose of 12 mg/kg & six times over 2 weeks; *i.p.*	↓Body weight, heart weight, and ratio of heart weight to body weight, ↑serum creatine kinase and LDH levels, ↑apoptosis, ↑p53 expression, ↑BAX protein, ↓Bcl-2 protein, ↑caspase-3 activity, ↓HO-1 expression and enzymatic activity	10 mg/kg/day & 1 week prior to DOX injection until two weeks after the last DOX injection (end of treatment); gavage	↑Body weight, heart weight, and ratio of heart weight to body weight, ↓serum creatine kinase and LDH levels, ↓apoptosis, ↓p53 expression, ↓BAX protein, ↑Bcl-2 protein, ↓caspase-3 activity, ↑HO-1 expression and enzymatic activity	Gu et al., 2012 [48]
In vitro/neonatal rat ventricular cardiomyocytes	1 *μ*M & 18 h	↑Cell death (↑PI-positive cells, cleaved caspase-3, and PARP), ↑autophagy (↑AV-positive cells, LC3-II, Atg5, and Atg5∗12, ↓p62, ↑phospho-S6K1, phospho-S6, and phospho-MBP)	10 *μ*M & 12 h prior to treatment	↓Cell death (↓PI-positive cells, cleaved caspase-3, and PARP), ↓autophagy (↓AV-positive cells, LC3-II, Atg5, and Atg5∗12, ↑p62, ↓phospho-S6K1, phospho-S6, and phospho-MBP)	Xu et al., 2012 [36]
In vivo/mice	8 mg/kg/week & for a total of 4 weeks; *i.p.*	↓Body weight, heart weight, and heart weight to tibia length ratio, ↑atrial natriuretic peptide, ↓SERCA2a, ↑ROS, ↓MnSOD, ↓mitochondrial electron transport chain complexes I and II	320 mg/kg/day & for 8 weeks; diet	↓Atrial natriuretic peptide, ↓ROS, ↑MnSOD, ↑mitochondrial electron transport chain complexes I, II, and IV, ↑mitofusin-1 and mitofusin-2 levels	Dolinsky et al., 2013 [50]
In vivo/mice	20 mg/kg & single dose; *i.p.*	Areas of myocytolysis with congestion of blood vessels, cytoplasmic vacuolization and fragmentation, hyalinization of muscle fiber, and chromatin margination of some nuclei, some pyknotic nuclei	Single dose of 15 mg/kg & cotreatment; *i.p.*	Normal muscle fibers with central oval nuclei and some pyknotic nuclei, fragmentation of the muscle fiber	Osman et al., 2013 [62]
In vivo/rats	12 mg/kg & single dose; *i.p.*	↑Edema and necrosis without normal myocardium	100 mg/kg & for 3 times (first one week before and the others with weekly intervals after DOX treatment); *i.p.*	↓Necrosis and ↑normal myocardium	Pınarlı et al., 2013 [63]
In vivo/rats	20 mg/kg & single dose; *i.p.*	↑Serum CPK and LDH enzymes, ↑MDA, ↓GSH, ↓TAC, massive fragmentation and lysis of myofibrils, vacuolization or complete loss of cristae, interruption of Z lines	10 mg/kg & cotreatment; *i.p.*	↓Serum CPK and LDH enzymes, ↓MDA, ↑GSH, ↑TAC, organized myofibrils with mitochondria in between, preservation of mitochondria structure similar to those of control group, observation of focal areas of myofibrillar loss and dilated sarcoplasmic reticulum	Al-Harthi et al., 2014 [54]
In vivo/rats	Accumulative dose of 15 mg/kg & 2.5 mg/kg in six injections for 2 weeks; *i.p.*	↓Body weight, absolute and relative heart weights, ↑CK-MB, ↑MDA, ↓GSH, ↓SOD, ↑hydroxyproline, ↑TNF-*α*, myocardial cell injury (necrosis, rupture of cardiac muscle fibers, myocyte wavy degeneration, massive interstitial hemorrhage, and interfibrillar congestion), ↑caspase-3 and TGF-*β*1 gene expression, ↑interstitial fibrosis, massive deposition of collagen	20 mg/kg/day & for 4 weeks (starting 2 weeks prior to DOX administration); gavage	↑Body weight, absolute and relative heart weights, ↓CK-MB, ↓MDA, ↑GSH, ↑SOD, ↓hydroxyproline, ↓TNF-*α*, moderate interstitial hemorrhage, necrosis, and fibrosis, ↓caspase-3 and TGF-*β*1 gene expression, ↓interstitial fibrosis	Arafa et al., 2014 [49]
In vitro/H9c2 cells	2 *μ*M & 12 h	↓Cell viability, ↑AMPK*α*2 and E2F1 expression, ↑cleaved PARP and caspase-3 levels	250 *μ*M & cotreatment	↑Cell viability, ↓AMPK*α*2 and E2F1 expression, ↓cleaved PARP and caspase-3 levels	Yang et al., 2014 [37]
In vitro/human cardiac progenitor cells and in vivo/rats	0.5 *μ*M & 24 h (for in vitro) and cumulative dose of 15 mg/kg & for 6 times with dose of 2.5 mg/kg over a period of 2 weeks; *i.p.* (for in vivo)	↓Body weight and ascites, ↓survival, ↓phospho-SIRT1^Ser27^, ↑ROS, ↑expression levels of acetyl-p53^Lys373^ and acetyl-p53^Lys382^, ↑apoptosis, ↓expression levels of IGF-1R and phospho-Akt^Ser473^, ↓cell viability, ↑senescence (↑p16^INK4a^ and *β*-galactosidase levels), ↑DNA damage, ↓migration ability	0.5 *μ*M & cotreatment (for in vitro) and 2.5 mg/kg/day & for 6 weeks (concomitantly with DOX administration and then were maintained for four more weeks); gavage (for in vivo)	↑Body weight and ascites, ↑survival, ↑SIRT1 expression, ↑phospho-SIRT1^Ser^ ^27^, ↑catalase and MnSOD, ↓ROS, ↓expression levels of acetyl-p53^Lys373^ and acetyl-p53^Lys382^, ↓apoptosis, ↑expression levels of IGF-1R and phospho-Akt^Ser473^, ↑cell viability, ↓senescence (↓p16^INK4a^ and *β*-galactosidase levels), ↓DNA damage, ↑migration ability	De Angelis et al., 2015 [38]
In vitro/H9c2 cells	5 *μ*M & 24 h	↓Cell viability, ↑apoptosis (↑GRP78 and CHOP expression), ↑SIRT1 level	25 *μ*M & for 24 h prior to DOX treatment	↑Cell viability, ↓apoptosis (↓GRP78 and CHOP expression), ↑↑SIRT1 level	Lou et al., 2015 [39]
In vivo/mice	20 mg/kg & single dose; *i.p.*	↑ROS, ↑apoptosis, ↓SIRT1, ↑cleaved caspase-3, ↑BAX, ↓Bcl-2, ↑phosphorylation-p38MAPK	10 mg/kg/day & for 8 days (3 days prior to DOX injection & 5 days after the injection); gavage	↓ROS, ↓apoptosis, ↑SIRT1, ↓cleaved caspase-3, ↓BAX, ↑Bcl-2, ↓phosphorylation-p38MAPK	Ruan et al., 2015 [59]
In vivo/mice	18 mg/kg & single dose on day 1; *i.p.*	↓Deacetylase activity of SIRT1, ↑protein content of p300, ↑acetylated Foxo1, ↑protein level of MuRF-1, ↑ubiquitinated proteins, ↑basal proteasomal activity and protein level of USP7, ↑p53, ↑BAX, ↑caspase-3 activity, ↑apoptotic DNA fragmentation	20 mg/kg/day & from day 2 to day 4; *i.p.*	↑Deacetylase activity of SIRT1, ↓protein content of p300, ↓acetylated Foxo1, ↓protein level of MuRF-1, ↓ubiquitinated proteins, ↓basal proteasomal activity and protein level of USP7, ↓p53, ↓BAX, ↓caspase-3 activity, ↓apoptotic DNA fragmentation	Sin et al., 2015 [60]
In vivo/mice	5, 5, and 15 mg/kg in days 2, 8, and 14; *i.p.*	↑Plasma LDH, ↓plasma catalase, GPx, GSH, and T-SOD, ↑plasma MDA, ↓activity of Ca2^+^-ATPase	200 *μ*mol/kg/day & for 15 days; intragastric	↑Plasma catalase and T-SOD	Wang et al., 2015 [61]
In vivo/rats	Cumulative dose of 15 mg/kg & 6 injections of 2.5 mg/kg over a period of 2 weeks; *i.p.*	↓Survival, ↑apoptotic myocytes and 8-OH-dG, ↑acetyl-p53^Lys381^ level, ↑myocardial collagen I mRNA expression and collagen I/III, ↓SIRT1 mRNA expression, ↑deacetylase activity of SIRT1, ↑mRNA level and protein expression of TGF-*β* and phospho-SMAD3^Ser423/425^/SMAD3 ratio, ↑*α*-SMA, FAP1*α*, CTGF, and MMP-2 levels, ↑gelatinolytic activity of MMP-2 in myocardium, ↑fibronectin	2.5 mg/kg/day & concomitantly with DOX administration and then were maintained for one more week; gavage	↑Survival, ↑catalase, MnSOD, and Cu/Zn-SOD, ↓apoptotic myocytes and 8-OH-dG, ↓acetyl-p53^Lys381^ level, ↓myocardial collagen I mRNA expression and collagen I/III, ↑SIRT1 mRNA expression, ↓deacetylase activity of SIRT1, ↓mRNA level and protein expression of TGF-*β* and phospho-SMAD3^Ser423/425^/SMAD3 ratio, ↓*α*-SMA, FAP1*α*, CTGF, and MMP-2 levels, ↓gelatinolytic activity of MMP-2 in myocardium, ↓fibronectin	Cappetta et al., 2016 [44]
In vitro/H9c2 cells and in vivo/rats	2 *μΜ* & 24 h (for in vitro) and 15 mg/kg/day & for 1 week; *i.p.* (for in vivo)	↑Apoptosis, ↓phospho-AMPK, ↑Beclin-1, ↑cleaved caspase-3, ↓Bcl-2, ↑BAX, ↑phospho-p53, ↑phospho-p38MAPK	20 *μΜ* & cotreatment (for in vitro)/10 mg/kg/day & cotreatment; *i.p.* (for in vivo)	↓Apoptosis, ↑phospho-AMPK, ↑LC3-II/LC3-I, ↑↑Beclin-1, ↓cleaved caspase-3, ↑Bcl-2, ↓BAX, ↓phospho-p53, ↓phospho-p38MAPK	Gu et al., 2016 [57]
In vitro/H9c2 cells	5 *μ*M & 24 h	↓Cell viability, ↓phospho-AMPK, ↑p53, ↑BAX, ↓Bcl-2, ↑apoptosis	25 *μ*M & 30 min prior to DOX treatment	↑Cell viability, ↑phospho-AMPK, ↓p53, ↓BAX, ↑Bcl-2, ↓apoptosis	Liu et al., 2016 [40]
In vitro/H9c2 cells	5 *μ*M & 24 h	↓Cell viability, ↑apoptosis, ↓SIRT1 level, ↑FoxO1, p53, and Bim levels, ↓SOD, ↑MDA, ↑LDH	25 *μ*M & 24 h prior to DOX treatment	↑Cell viability, ↓apoptosis, ↑SIRT1 level, ↓FoxO1, p53, and Bim levels, ↑SOD, ↓MDA, ↓LDH	Liu et al., 2016 [41]
In vitro/H9c2 cells	3 *μ*mol/L & for 24 h	↓Cell viability, ↑cleaved PARP and cleaved caspase-3	2 and 100 *μ*mol/L & NI	↑Cell viability, ↓cleaved PARP and cleaved caspase-3 (for 100 *μ*mol/L)	Yang et al., 2016 [42]
In vitro/neonatal rat cardiomyocytes	10 *μ*M & 6 h	↑Apoptosis	2.5 *μ*M or 250 nM (NI) & 48 h	No change on DOX-induced apoptosis	du Pré et al., 2017 [101]
In vivo/rats	Cumulative dose 2.5 mg/kg & six equal doses during 2 weeks; *i.p.*	↓Body weight, ↑NFAT3 level, ↓NFAT5 level, induction of histological changes (widely spaced deep acidophilic fibers, dense collagen fibers among thin fibers), ↑CK-MB and LDH, ↑BAX, ↓Bcl-xL, ↑caspase-3 expression	20 mg/kg/day & for 6 weeks (concomitantly with DOX administration for 2 weeks and then was continued for next 4 weeks); oral	↑Body weight, ↓NFAT3 level, ↑NFAT5 level, alleviation of DOX-induced histological changes (few congested blood vessels among muscle fibers, few deeply acidophilic, and few thin attenuated fibers, ↓fibrosis), ↓CK-MB and LDH, ↓BAX, ↑Bcl-xL, ↓caspase-3 expression	Shoukry et al., 2017 [51]
In vitro/H9c2 cells and in vivo/mice (normal and AMI mice)	1 *μ*M & 24 h (for in vitro) and cumulative dose of 20 mg/kg & 4 times over 4 weeks; *i.p.* (for in vitro & normal mice) and 5 mg/kg/day & for 7 days; *i.p.* (for in vitro & AMI mice)	↑Apoptosis, ↑E2F1, ↑mTORC1, ↓LC3-II/LC3-I, ↑AMPK*α*2, ↑cleaved caspase-3	20 *μ*M & cotreatment (for in vitro) and cumulative dose of 10 mg/kg/injection & prior to each DOX injection; *i.p.* (for in vitro & normal mice) and 10 mg/kg/day & for 7 days; *i.p.* (for in vitro & AMI mice)	↓Apoptosis, ↓E2F1, ↓mTORC1, ↑LC3-II/LC3-I, ↓AMPK*α*2, ↓cleaved caspase-3	Gu et al., 2018 [56]
In vivo/mice	4 mg/kg & once per week for 3 weeks; *i.p.*	↓Heart weight, ↑phospho-p38	0.4% of diet & for 4 weeks (starting one week prior to DOX injections and then was discontinued at one week after the last DOX injection); diet	↑Heart weight, ↓phospho-p38	Matsumura et al., 2018 [52]
In vivo/mice	20 mg/kg & single dose on day 4; *i.p.*	↓Body weight, ↓survival rate, induction of histological changes (distortion of the myocardial fibers and the cells with vacuole degeneration of various sizes)	20 mg/kg/day & for 9 days (4 days prior to DOX injection and then was continued for next 5 days); *i.g.*	↑Body weight, ↑survival rate, alleviation of DOX-induced histological changes	Zhang et al., 2019 [45]
In vivo/rats	Cumulative dose of 20 mg/kg & 2 mg/kg/injection and twice/week for 5 weeks (from weeks 2 to 6); *i.p.*	↑Serum CK-MB, troponin-I, and LDH levels, foci of degenerated myocardium, infiltration of inflammatory cells in the endomysium, ↑TLR-4, TNF-*α*, IL-6, and iNOS expression levels, ↑MDA, ↓GSH and SOD, ↓S100A1 and SERCA2a expression levels	20 mg/kg/day & for 6 weeks (starting one week prior to DOX administration); oral	↓Serum CK-MB, troponin-I, and LDH levels, foci of degenerated myocardium, ↓TLR-4, TNF-*α*, IL-6, and iNOS expression levels, ↓MDA, ↑GSH and SOD	Alanazi et al., 2020 [53]
In vitro/H9c2 cells and neonatal rat cardiomyocytes and in vivo/rats	1 *μ*mol/L & 24 h (for in vitro) and 10 mg/kg/injection & twice/week for 2 weeks (in the fifth and sixth weeks); *i.p.* (for in vivo)	↓Cell viability, ↓heart weight to body weight ratio, ↑serum LDH and CK-MB levels, ↑apoptosis, ↑BAX, ↓VEGF-B, phospho-Akt and phospho-GSK-3*β* expressions	50 *μ*mol/L & 48 h prior to DOX treatment+50 *μ*mol/L & cotreatment (for in vitro) and 50 mg/kg/day & for 6 weeks; gavage (for in vivo)	↑Cell viability, ↑heart weight to body weight ratio, ↓serum LDH and CK-MB levels, ↓apoptosis, ↓BAX, ↑VEGF-B, phospho-Akt and phospho-GSK-3*β* expressions	Tian et al., 2020 [43]

↑: increase; ↓: decrease; NI: not informed; i.p.: intraperitoneal; i.g.: intragastrical; DOX: doxorubicin; MDA: malondialdehyde; ROS: reactive oxygen species; GPx: glutathione peroxidase; SOD: superoxide dismutase; MMP-2: matrix metalloproteinase-2; PARP: poly (ADP-ribose) polymerase; BAX: Bcl-2-associated X protein; Bcl-xL: B-cell lymphoma-extra large; IL-6: interleukin 6; TNF-*α*: tumor necrosis factor alpha; LDH: lactate dehydrogenase; FABP: fatty acid binding protein; BNP: brain natriuretic peptide; AST: aspartate aminotransferase; ALP: alkaline phosphatase; PI: propidium iodide; S6K1: p70 S6 kinase 1; SERCA2a: sarcoplasmic/endoplasmic reticulum calcium-ATPase 2a; MnSOD: manganese superoxide dismutase; CPK: creatine phosphokinase; GSH: glutathione; CK-MB: creatine kinase-myocardial band; AMI: acute myocardial infarction; MPO: myeloperoxidase; T-SOD: total superoxide dismutase; SIRT1: sirtuin 1; TBARS: thiobarbituric acid reactive substances; HO-1: heme oxygenase-1; CPK: creatine phosphokinase; TGF-*β*1: transforming growth factor beta 1; TAC: total antioxidant capacity; IGF-1R: insulin-like growth factor 1 receptor; NFAT3: nuclear factor of activated T cells 3; mTORC1: mammalian target of rapamycin complex 1; AMPK: adenosine monophosphate- (AMP-) activated protein kinase; VEGF-B: vascular endothelial growth factor B; iNOS: inducible nitric oxide synthase; TLR-4: toll-like receptor-4.

## Data Availability

The data used to support the findings of this study are available from the corresponding author upon request.
